# PARP Traps Rescue the Pro-Inflammatory Response of Human Macrophages in the In Vitro Model of LPS-Induced Tolerance

**DOI:** 10.3390/ph14020170

**Published:** 2021-02-22

**Authors:** Julita Pietrzak, Karolina Gronkowska, Agnieszka Robaszkiewicz

**Affiliations:** Department of General Biophysics, Faculty of Biology and Environmental Protection, University of Lodz, Pomorska 141/143, 90-236 Lodz, Poland; julita.pietrzak@unilodz.eu (J.P.); kar.gronkowska@gmail.com (K.G.)

**Keywords:** immunoparalysis, lipopolysaccharide tolerance, macrophages, sepsis, poly(ADP-ribose) polymerase-1 (PARP1)

## Abstract

Secondary infections cause sepsis that lead to patient disability or death. Contact of macrophages with bacterial components (such as lipopolysaccharide—LPS) activates the intracellular signaling pathway downstream of Toll-like receptors (TLR), which initiate an immune proinflammatory response. However, the expression of nuclear factor-kappa B (NF-κB)-dependent proinflammatory cytokines significantly decreases after single high or multiple LPS stimulations. Knowing that poly(ADP-ribose) polymerase-1 (PARP1) serves as a cofactor of NF-κB, we aimed to verify a hypothesis of the possible contribution of PARP1 to the development of LPS-induced tolerance in human macrophages. Using *TNF-α* mRNA expression as a readout, we demonstrate that PARP1 interaction with the *TNF-α* promoter, controls macrophage immunoparalysis. We confirm that PARP1 is extruded from the gene promoter, whereas cell pretreatment with Olaparib maintains macrophage responsiveness to another LPS treatment. Furthermore, cell pretreatment with proteasome inhibitor MG132 completely abrogates the effect of Olaparib, suggesting that PARP1 acts with NF-κB in the same regulatory pathway, which controls pro-inflammatory cytokine transcription. Mechanistically, PARP1 trapping allows for the re-rebinding of p65 to the *TNF-α* promoter in LPS-stimulated cells. In conclusion, PARP traps prevent PARP1 extrusion from the *TNF-α* promoter upon macrophage stimulation, thereby maintaining chromatin responsiveness of TLR activation, allowing for the re-binding of p65 and *TNF-α* transcription.

## 1. Introduction

Sepsis-induced immunoparalysis is a complication of secondary infections that compromises the immune response [1]. It is a life-threatening dysfunction caused by a dysregulation of the host response to infection. It leads to death or disability of patients and costs to the healthcare system [2,3]. Immune response in sepsis is characterized by the excessive pro-inflammatory response to pathogen-associated molecules, such as endotoxin (e.g., lipopolysaccharide (LPS) from *Escherichia coli*), with a simultaneous decreasing anti-inflammatory mechanism. Many models, including in vitro macrophage cultures, reveal that multiple LPS stimulation induces attenuation of pro-inflammatory response. This phenomenon is known as “endotoxin tolerance” [2,4]. The contact of bacterial LPS with the Toll-like receptor 4 (TLR4) on the macrophage cell surface induces secretion of numerous proinflammatory cytokines and factors, such as tumor necrosis factor-α (TNF-α); interleukins IL-1β, IL-6, and IL-8; macrophage inflammatory protein 2 (MIP2); and cyclooxygenase-2 (COX2) [5,6].

Stimulation of TLR4 activates the nuclear factor-kappa B (NF-κB)/mitogen signaling pathway, which involves several adaptor proteins, including myeloid differentiation factor 88 (MyD88), IL-1 receptor-associated kinase (IRAK), and tumor necrosis factor-alpha receptor-associated factor 6 (TRAF6), and thus leads to transcription of NF-κB-dependent pro-inflammatory cytokines [7,8]. NF-κB transcription factor consists of few subunits—p65 (RelA), RelB, c-ReL, p50 (NF-κB1), and p52 (NF-κB2)—that form homo- or heterodimers in the cytoplasm. The phosphorylation and degradation of inhibitor of κB proteins (IκB) in the canonical allow for the translocation of the NF-κB dimers into the nucleus, where they activate transcription as heterodimers (p50–p65) or repress transcription as homodimers. Moreover, p50 homodimers repress the inflammation by recruitment of co-repressors, such as histone deacetylases (HDACs), which remove acetyl groups from histones, thereby leading to chromatin condensation and transcription repression [9].

Transcription activation by canonical NF-κB heterodimers often requires cofactors, such as poly(ADP-ribose) polymerase-1 (PARP1). The enzyme belongs to the PARP family that comprises 17 members, which vary in the mode of protein mono- and poly-ADP-ribosylation, subcellular location, as well as in cellular function. A growing number of processes has been shown to be regulated by PARP1, which covalently modifies its targets by utilizing nicotinamide adenine dinucleotide (NAD) to synthesize poly-ADP-ribose polymers as well as physically interacts with various proteins and DNA, thereby tuning their activity, structure, or biological function [10]. By the interaction with p300 acetyltransferase, PARP1 coactivates NF-κB-dependent gene expression in response to single LPS stimulation. It also promotes the formation of the preinitiation complex at the gene promoters in the transcription-factor-binding regions [11,12]. This enzyme was postulated to contribute to the development of inflammatory disorders, such as rheumatoid arthritis, diabetes, neurodegenerative disorders (including Parkinson’s and Alzheimer’s), infarction–reperfusion, and septic shock. Numerous recent scientific reports suggest that PARP inhibition serves as a promising strategy to treat immune diseases [13,14]. Some PARP inhibitors, including olaparib, act as PARP1–DNA traps at the sites off single-strand breaks, and prevent the repair of (and enhance) the generation of the double-strand breaks, which are particularly detrimental for breast cancer 1/2 (BRCA1/2)-deficient tumors [15]. In this study, we demonstrate that PARP1 trapping on the chromatin with olaparib in LPS-stimulated cells may have a new application in maintaining an NF-κB-dependent pro-inflammatory response.

## 2. Results

### 2.1. PARP Trapping Prior to Macrophage Activation Maintains Their Pro-Inflammatory Response

**Figure 1:**
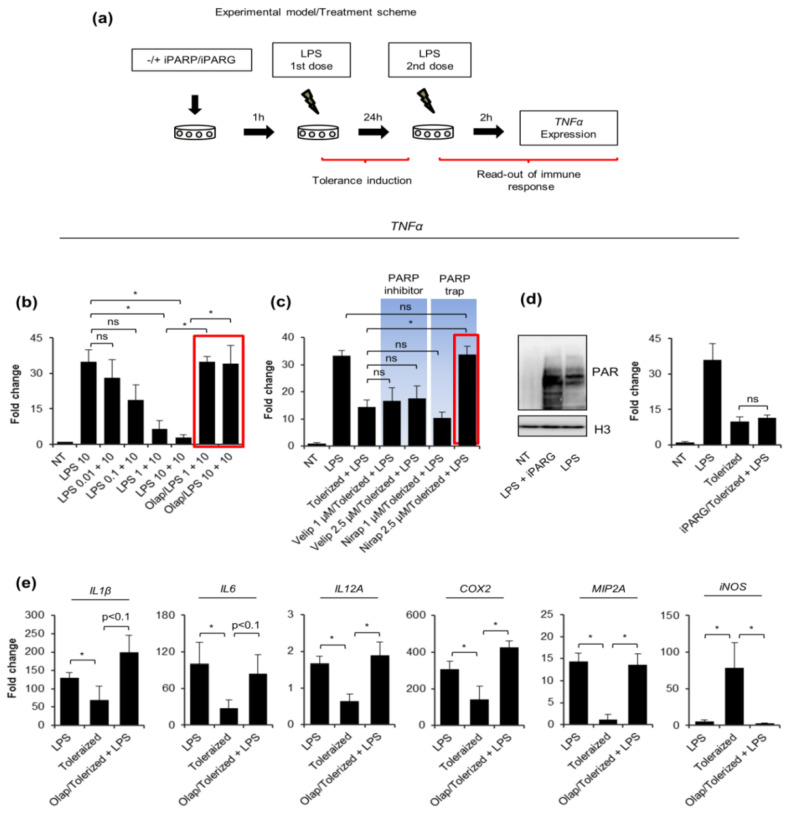
PARP traps maintain proinflammatory response in human macrophages activated with tolerance-inducing LPS doses. (**a**) The scheme of tolerance induction presents an experimental approach and cell treatments: the first dose of LPS aims to induce cell paralysis or priming within 24 h, the second serves to check macrophage pro-inflammatory response using mRNA of TNF-α as a readout after cell stimulation for 2 h. To test a possible effect of the key enzymes involved in the ADP-ribosylation metabolism (PARP inhibitors: olaparib, veliparib, niraparib, and PARG inhibitor: ADP–HPD) on the tolerance development, the corresponding compounds were added for 1 h prior to the tolerance-inducing (first) dose of bacterial endotoxin. Expression of TNF-α was quantified by real-time PCR, normalized to median of ACTB and GAPDH, and shown as a fold change with respect to control cells (LPS untreated = 1). (**b**) Three doses of LPS were tested for macrophage paralysis and the immunomodulatory effect of PARP inhibitor—olaparib (1 µM) was estimated based on the TNF-α transcription. The red rectangular indicates the couple: olaparib-LPS that was chosen for further experiments. (**c**) The other two PARP inhibitors, which differ in PARP-DNA binding ability—niraparib (MK-4827) and veliparib (ABT-888), as well as (**d**–**e**) PARG inhibitor—ADP–HPD (10 µM) were analyzed for their possible effect on the induction of tolerance by LPS. (**d**) The increased accumulation of ADP-ribosylated proteins caused by ADP–HPD pretreatment of LPS-induced macrophages was confirmed by western blot. H3 was used as a loading control. Full-length western blot images are included in the Supplementary Figure S3. (**e**) The possible modulatory role of PARP1 in the paralysis of macrophage pro-inflammatory phenotype was tested also for other cytokine genes, such as IL-1β, IL-6, IL-12, MIP2A, COX2, and iNOS using real-time PCR for the measurement of mRNA levels. Bars in the figures represent mean ± standard error of the mean (SEM). One-way analysis of variance (ANOVA1) was carried out in GraphPad Prism 5 to compare means in several groups. Once the significance was detected, ANOVA1 was followed by the Tukey post-hoc test and significant differences between the two considered means are marked with * when significant at *p*  <  0.05, ns—non-significant at *p* > 0.05. Abbreviations: iPARP—poly(ADP-ribose) polymerase (PARP) inhibitor(s), iPARG—poly(ADP-ribose) glycohydrolase (PARG) inhibitor, LPS—lipopolysaccharide, Olap—olaparib, Velip—veliparib, Nirap—niraparib, TNF—tumor necrosis factor, IL1β—interleukin 1 beta, IL6—interleukin 6, IL-12A—interleukin 12 subunit alpha, COX2—cyclooxygenase-2, MIP2A—macrophage inflammatory protein 2 subunit alpha, iNOS—inducible nitric oxide synthase, ACTB—actin beta, GAPDH—glyceraldehyde 3-phosphate dehydrogenase.

To test the possible contribution of PARP1 to the development of endotoxin-induced tolerance of human macrophages, we first optimized the doses of LPS, which paralyze the pro-inflammatory response of considered phagocytes. We used transcription of *TNF-α* (mRNA) as a readout and set a 24 h window between two consecutive cell treatments with LPS (Figure 1a). As shown in Figure 1b, two higher concentrations of LPS substantially blocked the expression of TNF-α, thereby indicating the development of macrophage tolerance to endotoxin. For these two concentrations, we checked the immunomodulatory potential of olaparib, which acts as a PARP1–DNA trap, inhibits PARP activity, and prevents protein ADP-ribosylation. Macrophage pretreatment with 1 µM olaparib for 1 h prior to tolerance induction maintained the pro-inflammatory response of phagocytes (Figure 1b). Due to the observed higher variation of TNF-α expression in macrophages stimulated with 10 ng/mL LPS, and its lower biological relevance, we chose 1 ng/mL LPS for the induction of immune paralysis in the following experiments. In contrast to macrophages, human monocytes that are PARP1-deficient did not respond to olaparib and showed tolerance to bacterial endotoxin regardless of their pretreatment with the PARP inhibitor (Supplementary Figure S1) [16].

To distinguish between the possible effect of ADP-ribosylation and PARP trapping on the tolerance development, we involved another two PARP inhibitors that differ in the DNA-binding potential. Olaparib and niraparib are far more effective in PARP trapping than veliparib at the concentrations where they fully inhibit PARylation [14,17]. Moreover, we tested two concentrations for each of the two compounds. None of the studied PARP inhibitors increased the *TNF-α* transcription under the LPS-free condition (Supplementary Figure S2). Results in Figure 1c indicate the anti-paralyzing potential of Niraparib, which was particularly prominent at the higher dose. The lack of impact of veliparib on *TNF-α* transcription in tolerized cells provides further evidence that the interaction of some PARP family members (PARP1, PARP2, PARP3) with the chromatin, rather than ADP-ribosylation, somehow contributes to macrophage paralysis. To further support such a hypothesis and confirm that ADP-ribosylation does not promote the tolerance to endotoxin, the mRNA of *TNF-α* was quantified in cells deficient in the activity of poly-ADP-ribose-degrading enzyme poly-(ADP-ribose) glycohydrolase (PARG). As shown in Figure 1d,e, the accumulation of poly-ADP-ribose polymers due to the pretreatment of macrophages with PARG inhibitor ADP–HPD did not alter *TNF-α* transcription.

Since pro-inflammatory response involves numerous other cytokines in addition to TNF-α, we checked the profile of *IL1β*, *IL6*, *IL12A*, *MIP2A*, *COX2*, *iNOS* transcription under the same experimental conditions in differentiated human macrophages (Figure 1e). Most of them, but *iNOS*, showed considerable decline in responsiveness after the first stimulation with 1 ng/mL LPS. As same as for *TNF-α,* the applied PARP trap maintained or even increased the cytokine transcription after the second dose of bacterial endotoxin when compared to 10 ng/mL LPS alone.

These results all indicate that the PARP occurrence on chromatin during the first challenge with LPS rescues the pro-inflammatory phenotype of human macrophages.

### 2.2. PARP1 Level Serves as a Key Determinant of Tolerance Development

Since, among other PARP family members, PARP1 has been most frequently linked to canonical NF-κB signaling and documented as inflammatory-relevant factor, we first paid attention to this enzyme. Due to the lack of commercially available and specific PARP1 inhibitors, we decided to make use of both silencing and overexpression of the enzyme. Bearing in mind that mRNA targeting in phagocytes is of a considerable challenge and that cationic transfection reagents uncouple LPS binding by preventing CD14–TLR4 interactions (we failed to induce tolerance to LPS in chemically transfected human macrophages), we changed macrophages to THP1 human monocytic cell line to generate stable PARP1 knockdowns [18].

**Figure 2:**
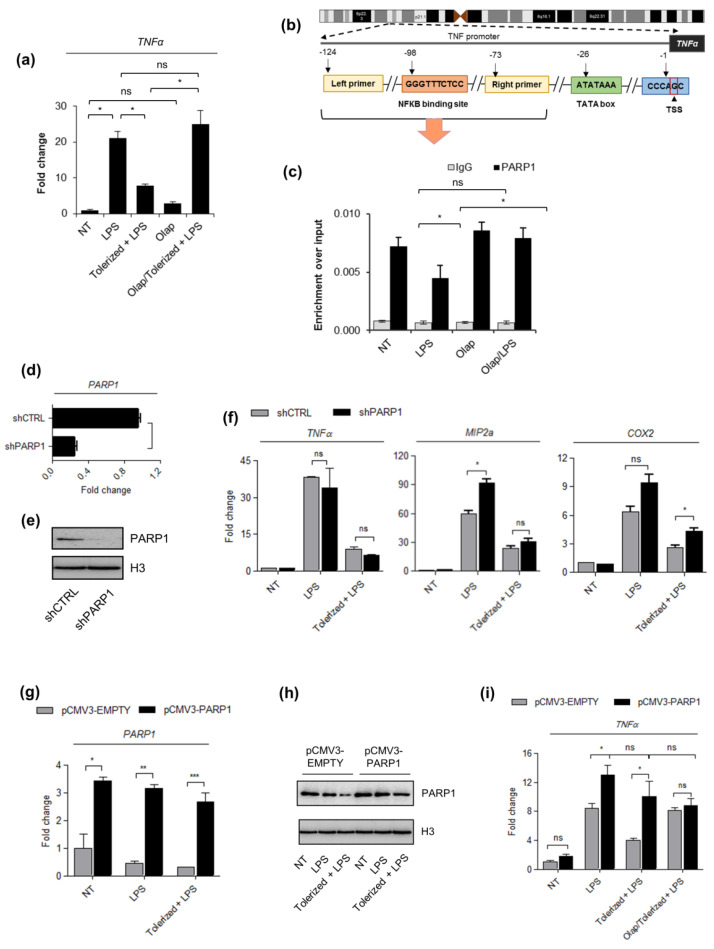
The PARP1 level drives LPS-induced immune paralysis in THP1 cells. (**a**) The induction of tolerance in acute leukemia cells was evaluated based on the *TNF-α* expression that was quantified by real-time PCR, normalized as median of *ACTB* and *GAPDH*, and shown as a fold change with respect to untreated cells. (**b**) The illustration in outlines the human proximal *TNF-α* promoter and the position of NF-κB (−98) as well as primer (forward: −124, reverse: −73) binding sites with respect to transcriptional start site (TSS). The fragment between the two indicated primers was amplified to quantify the immunoprecipitated DNA by real-time PCR. (**c**) The impact of the single LPS dose and olaparib on PARP1 occurrence at the *TNF-α* promoter was estimated with ChIP-qPCR. (**d**–**e**) The silencing efficiency of PARP1 in THP1 cells stably transfected with PARP-1 shRNA Plasmid was determined by real-time PCR and western blot, respectively versus corresponding control cells (shCTRL; control shRNA Plasmid-A). *PARP1* mRNA was normalized to the median of *ACTB* and *GAPDH*, and shown as a fold change with respect to control cells with the normal PARP1 expression. In western blot, H3 was used as a loading control. Full-length western blot images are available in the Supplementary Figure S4. (**f**) The mRNA level of *TNF-α*, *MIP2a*, and *COX2*, which served to assess the development of immune tolerance in response to LPS, was measured with real-time PCR as described above. (**g**–**h**) PARP1 overexpression in pCMV3–PARP1 versus pCMV3–EMPTY-transfected cells was confirmed by real-time PCR and western blot, respectively. Full-length western blot images are included in the Supplementary Figure S5. mRNA level of PARP1 was normalized to *ACTB* and *GAPDH* and is presented as a fold change of PARP1 knock-in with respect to control cells. (**i**) The effect of PARP poison (olaparib) on the development of endotoxin tolerance in pCMV3–EMPTY and pCMV3–PARP1 cells was estimated on the basis of *TNF-α* transcription (mRNA), which was set as a readout. Bars in the figures represent mean ± standard error of the mean (SEM). One-way analysis of variance (ANOVA1) was carried out in GraphPad Prism 5 to compare means in several groups. Once the significance was detected, ANOVA1 was followed by the Tukey post-hoc test and significant differences between the two considered means are marked with * when significant at *p*  *<*  0.05, ** when significant at *p*  *<*  0.01, *** when significant at *p*  *<*  0.001, ns—non-significant at *p* > 0.05. Abbreviations: THP1—human monocytic cell line, LPS—lipopolysaccharide, Olap—Olaparib, TNF—tumor necrosis factor, ACTB—actin beta, GAPDH—glyceraldehyde 3-phosphate dehydrogenase, shCTRL—THP1 cell transfected with Control shRNA Plasmid-A, shPARP1—THP1 cell transfected with PARP-1 shRNA Plasmid, pCMV3–PARP1—THP1 cells transfected with pCMV3–-PARP1 plasmid, pCMV3–EMPTY—THP1 cells transfected with pCMV3-EMPTY plasmid, IgG—immunoglobulin G.

First, we tested these cells as a model to study the principles of tolerance induction to LPS and checked whether PARP trapping also protects these cells from LPS-induced tolerance. Results in Figure 2a indicate that THP1 cells develop resistance to LPS and stop responding to the consequent dose of bacterial endotoxin. Similar to macrophages, olaparib protects *TNF-α* from LPS-triggered repression. Knowing that PARP1 is assigned to the synthesis of over 80% of ADP-ribose polymers and is frequently documented as a transcription coregulator, we first followed the PARP1 occurrence at the *TNF-α* promoter in intact cells and cells challenged with bacterial endotoxin. We searched for PARP1 in the region overlapping the NF-κB-binding site (Figure 2b) since this enzyme was documented in various contexts as a cofactor of NF-κB-dependent transcription of cytokines in macrophages. The results of ChIP-qPCR confirmed the occurrence of PARP1 at the *TNF-α* promoter and the extrusion of the enzyme, as soon as 1 h after LPS treatment (Figure 2c). Olaparib, which is of relatively high trapping potency, maintained PARP1 on the chromatin. In summary, Olaparib prevents PARP1 extrusion from *TNF-α* promoter in response to LPS, and maintains TNF-α responsiveness to bacterial endotoxin.

If PARP1 extrusion from the *TNF-α* promoter contributes to the development of endotoxin tolerance, then PARP1 deficiency was expected to have no effect on or enhance *TNF-α* suppression. To verify this hypothesis, we generated stably silenced PARP1 knockdowns by THP1 transfection with shPARP1-carrying versus shCTRL vector and selection with puromycin (Figure 2d,e). As expected, the considerable decrease in PARP1 abundance did not interfere with macrophage paralysis (Figure 2f). The analysis extended to two other pro-inflammatory cytokines, *COX2* and *MIP2a,* LPS-induced suppression of which was also prevented by olaparib in human macrophages, led to the same conclusion.

In such a case, we took a reverse approach and transiently overexpressed PARP1 in THP1 cells by their transfection with PARP1-overexpressing vector. We made use of ViaFect Transfection Reagent and an optimized transfection protocol, which allowed us to induce tolerance in THP1 transfected cells. As documented in our previous study [19], LPS caused repression and cleavage of PARP1, whereas overexpression of the enzyme maintained high PARP1 abundance regardless of LPS treatment (Figure 2g,h). PARP1 overexpression protected cells from *TNF-α* repression induced by bacterial endotoxin. This all confirms that LPS-induced PARP1 deficiency at the *TNF-α* promoter allows for gene silencing and inhibits gene responsiveness to another cell stimulation with LPS (Figure 2i).

### 2.3. PARP1 Extrusion form TNF-α Promoter Hampers p65 Rebinding after Tolerance Induction

**Figure 3:**
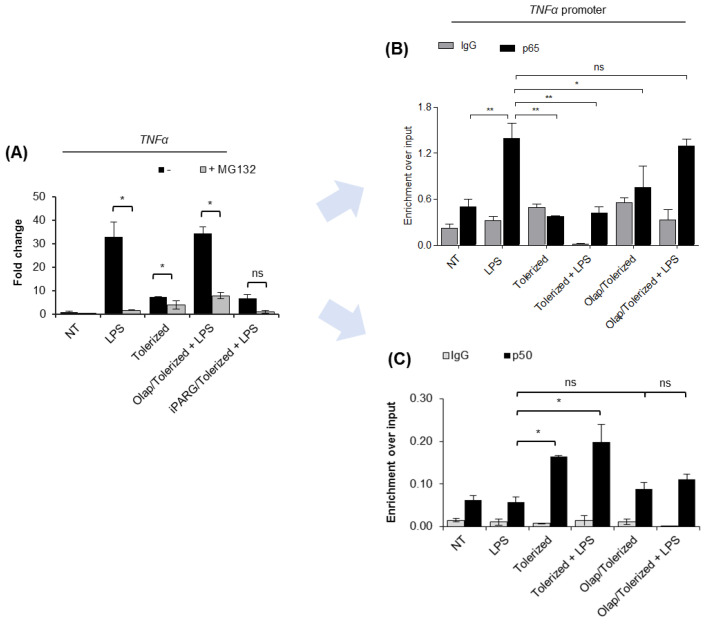
PARP1 eviction from the *TNF-α* promoter prevents p65 re-binding after LPS re-stimulation. (**A**) The contribution of canonical NF-κB to macrophage response to LPS was estimated by comparing *TNF-α* transcription in control and cells pre-treated with proteasome inhibitor (MG132; 1 µM). mRNA level of the cytokine was quantified by real-time PCR, normalized to median of *ACTB* and *GAPDH* and presented as a fold change with respect to untreated cells. (**B**) p65 and (**C**) p50 occurrence at the *TNF-α* promoter was analyzed by ChIP-real-time PCR. Bars in the figures represent mean ± standard error of the mean (SEM). One-way analysis of variance (ANOVA1) was carried out in GraphPad Prism 5 to compare means in several groups. Once the significance was detected, ANOVA1 was followed by the Tukey post-hoc test and significant differences between the two considered means are marked with * when significant at *p*  <  0.05, ** when significant at *p*  *<*  0.01, ns—non-significant at *p* > 0.05. Abbreviations: LPS—lipopolysaccharide, Olap—olaparib, TNF—tumor necrosis factor, ACTB—actin beta, GAPDH—glyceraldehyde 3-phosphate dehydrogenase, IgG—immunoglobulin G, p65—transcription factor p65 (RelA), p50—nuclear factor NF-kappa-B p105 subunit.

To confirm that PARP1 operates on chromatin in the same regulatory circuit with canonical NF-κB (p65-p50 heterodimers), we first estimated the degree of NF-κB contribution to macrophage response to LPS. Cell pretreatment with proteasome inhibitor MG132, which protects IκB (NF-κB inhibitor) from ubiquitination and proteasomal degradation, therefore precludes NF-κB binding to the target gene promoters, completely blocked LPS-induced transcription of *TNF-α* (Figure 3A). The effect of PARP inhibitor olaparib was abrogated in the presence of MG132, suggesting that PARP1 affects NF-κB-dependent transcription of pro-inflammatory cytokine.

Analysis of the occurrence of p65 at the *TNF-α* promoter revealed the immediate binding of this transcription factor after cell stimulation with LPS and its complete loss in tolerized cells (Figure 3B). It suggests that the initial wave of p65 recruitment to chromatin is transient, followed by the extrusion of the protein from the studied gene promoter. Stimulation of tolerized cells with another dose of LPS did not result in p65 rebinding. Importantly, PARP1 trapping at the promoter of *TNF-α* maintained chromatin responsiveness and allowed for p65 recruitment in tolerized cells. Notably, the extent of p65 enrichment in cells tolerized in the presence of olaparib was comparable to that of intact cells challenged for the first time with bacterial endotoxin. These results suggest that PARP1 extrusion from the gene promoter during the first contact of cells with LPS allows for chromatin remodeling, which impedes further recruitment of p65.

Knowing that p50 forms transcription-promoting heterodimers with p65 and that enrichment of p50 in certain circumstances leads to gene suppression due to the assembly of repressive complexes with chromatin remodeling enzymes such as HDAC1, we tested the occurrence of p50 at the promoter of *TNF-α* in intact and tolerized cells. As shown in Figure 3C, the single and lower dose of LPS (10 ng/mL) did not trigger a considerable enrichment of p50 at the promoter of *TNF-α.* It might be surprising in the light of well-approved concept of p65–p50 heterodimer contribution to transcriptional gene activation, and it may indicate the preexistence of certain pool of p50 at the NF-κB-dependent gene promoters that is necessary for immediate gene response after recruitment of p65. Certainly, such an aspect needs to be taken into further consideration. Anyway, the substantial enrichment of p50 was observed 24 h after tolerance induction and remained unchanged after the second dose of LPS. PARP1 trapping on chromatin with olaparib prior to LPS prevented p50 enrichment in tolerized cells. This indicates that PARP1 extrusion from the promoter of *TNF-α* in response to the first contact of cell with LPS allows for p50 interaction with the gene promoter, which might possibly preclude the binding of p65 in tolerized cells by recruiting other repressors and, thereby, chromatin remodeling [9]. Again, such a hypothesis requires further and mechanistic examination.

## 3. Discussion

Sepsis is the outcome of the invalid coordination of inflammation against pathogens driven by monocytes and macrophages as well as other granulocytes, which belong to the first line of immune defense [20]. Long-term or high doses of lipopolysaccharide exposure of these cells can lead to endotoxin tolerance, which is seen in patients with sepsis. LPS paralyzes the proper immune response and reprograms it by reducing the production of pro-inflammatory cytokines such as TNF-α, IL-1β, IL-6, and others [21,22]. Many studies have described the impact of LPS-induced tolerance on the development of sepsis. Nonetheless, the correlation between the immunoparalysis and epigenetic modifications of cytokine regulatory elements is still under investigation. High-dose LPS stimulation of monocytic cells contributes to the decreased nuclear level of p65 and its binding to DNA, with simultaneous activation of p50 homodimers and their recruitment to chromatin. This can represent an adaptive response to prevent the harmful consequences of excessive production and release of cytokines such as TNF-α [23,24]. Stimulation of macrophage with bacterial ligand of TLRs triggers gene reprogramming, mainly by epigenetic changes, such as nucleosome and chromatin remodeling, histone modifications, and reduction of transcription factor recruitment [25]. For example, NF-κB can recruit nuclear receptor co-repressor 1–histone deacetylase 3–p50 (NCOR-HDAC3-p50) repressive complex or methyltransferase G9a to promoters, thereby leading to epigenetic silencing [26,27]. Particularly, in the described murine genome, p50 emerged essential for assembling the repressosome and LPS-induced tolerance. In that experimental model, the enrichment of HDAC1, HDAC3, and NcoR negatively correlated with the occurrence of p65 at the promoter of *TNF-α* [26]. The deacetylation of nucleosomes by HDAC1 and/or HDAC3 possibly prevent the recruitment of p65. Earlier study on NF-κB p65 subunits provided evidence on their binding to genes characterized higher levels of histone acetylation and that increase in aforementioned, transcription promoting modification precedes the p65 interaction with DNA [28]. Moreover, p50 homodimers, which bind nucleosomal κB sites in vitro, were also observed in the nucleus of unstimulated cells, and were displaced by activating dimers (p65 or c-Rel) in stimulated cells, possibly due to the chromatin remodeling. Therefore, the observed enrichment of p50 at the promoter of *TNF-α* after the first macrophage stimulation with LPS may be associated with formation of repressive complexes, which deacetylate nucleosome, thereby making chromatin inaccessible for p65. LPS-tolerized macrophages were shown unable to deposit active histone marks at promoters in response to LPS restimulation. Moreover, LPS-treated macrophages maintain the epigenetic profile of chromatin similar to monocytes, which is low in H3K4me1 and high in H3K27me3 occurrence [29,30]. A previously published report indicates that the dimer composition of the NF-κB complex, which is recruited or occupies the gene promoters, varies in LPS-responsive and LPS-tolerized cells [31]. As described in Porta et al., the tolerance is followed by the extensive occurrence of p50 homodimer rather than p65–p50 heterodimers [32]. A similar situation was also found in tumor-associated macrophages (TAMs). This agrees with our study, where LPS-trained macrophages were characterized by the considerable enrichment of p50 at the promoter of *TNF-α*. Moreover, our results provide the first evidence that the PARP1 enrichment at the gene promoter of *TNF-α* prevents LPS-induced switching from transcription-promoting p65–p50 to repressive p50–50 dimers. The brake on the NF-κB-dependent inflammatory gene transcription may be in part mediated by an interaction of p50 with the epigenetic repressor protein histone deacetylase 1 (HDAC1), which is capable of removing nucleosome acetylation, and by the lack a transcription activation domain in p50 [33].

In macrophages, PARP1 facilitates inflammatory cytokine expression by promoting NF-κB accessibility at regulatory sequences [22]. According to our findings, macrophage response to higher doses of LPS leads to a substantial decrease in NF-κB-dependent expression of TNF-α. The product of this gene is considered as a marker of inflammatory macrophage response and, thus, also of endotoxin tolerance. The macrophage pretreatment with olaparib prior to LPS stimulation maintains a pro-inflammatory response. It confirms the role of PARP1 in cytokine expression control that has already been published by other groups [31,34]. Furthermore, the involvement of PARP inhibitors that differ in the DNA binding potential showed that PARP1 extrusion from the chromatin allows for chromatin remodeling, which prevents p65 rebinding upon the following macrophage stimulation with LPS. PARP1 occurrence at the *TNF-α* promoter prevents enrichment of p50, which was observed upon macrophage primary stimulation with LPS. Importantly, PARP trapping at the gene promoter, but not inhibition of ADP-ribosylation, turned out to protect macrophages from endotoxin-induced tolerance. Further investigation of mechanical aspects of the negative correlation between PARP1 and p50 upon activation of TLR-downstream signaling pathways is needed to explain the observed phenomenon.

PARP1 extrusion from the chromatin is induced by the activation of TLR receptors. TLR activation is essential for innate response against pathogens; nonetheless, the same group of receptors may contribute to the development of the chronic inflammatory state that is characteristic of sepsis [35,36]. Therefore, PARP trapping may serve as an alternative to TLR antagonists, which have been tested in clinical trials. These include Lipid A derivatives (glycolipids) and anti-TLR4 antibody or TLR4 ligand-J KB-122, which has been approved by the Food and Drug Administration (FDA) for the treatment of autoimmune hepatitis (AIH) [37]. TLR downstream targets include adaptor molecules and kinases (such as MyD88, IRAK, or TRAF6) and NF-κB transcription factor, which have been documented as active players in the development of endotoxin tolerance [38]. Therefore, inhibition of poly-ADP-ribosylation may also inhibit NF-κB pathways at these steps, which require PARP1 activity. ADP-ribose molecules were shown to be crucial for the formation of ATM-PIASy-IKKg that led to IKK proteasomal degradation and NF-κB activation upon DNA damage, which also occurs during cell stimulation with LPS [39]. This suggests that interference with the ADP-ribosylation can possibly modulate the NF-κB pathway responsible for the development of endotoxin tolerance at more than one-step.

Due to the limited possibility of genetic manipulation in macrophages, we performed some experiments on a THP1 human monocytic cell line. Although cancer cells differ from primary macrophages in inter alia expression of LPS receptor CD14, they responded similarly to LPS also in terms of PARP1 contribution to the induction of endotoxin tolerance [40]. This indicates that the PARP1 role in LPS-triggered *TNF-α* transcription is similar among various (myeloid) cell types [41]. It is also of particular importance for anti-cancer therapies, which involve PARP inhibitors, since these compounds may help to prevent macrophage reprogramming to a trained phenotype that resembles M2 macrophages. On the other hand, the M2d macrophage subtype that has been linked with the tumor progression is characterized by the extensive production of TNF-α, and PARP poisons may facilitate tumor progression by promoting the activity of tumor-associated macrophages (TAMs) [42]. In any case, further in vivo validation of PARP traps is necessary to confirm the immunomodulatory effects of PARP1 binding to DNA.

Sepsis is a highly lethal disease entity that, according to a recent scientific publication in 2017, had 48.9 million cases and 11 million sepsis-related deaths worldwide, which constituted almost 20% of all global deaths [43]. The success of treatment depends on two factors considered in the medical environment: an early and appropriate antibiotic, as well as all-purpose supporting treatment [44]. In recent years, numerous studies have been provided, which described the approaches of sepsis patient treatment. These include TNF-α neutralizing antibodies, bactericidal/permeability-increasing protein or platelet-activating factor, with a positive result in intensive care units [45]. The ongoing clinical trials have been testing anti-CD14 antibodies or lipid A analogs as antiendotoxin agents [46]. Our study describes another option to prevent the development of sepsis: the use of PARP traps, which are capable of binding PARP1 to chromatin, to maintain the macrophage pro-inflammatory phenotype upon succeeding stimulation of phagocytes with LPS. Due to the contribution of PARP1 to DNA repair, PARP inhibitors have been extensively tested, and some of them (as well as PARP traps, including olaparib and talazoparib) have been approved for the monotherapies of patients with breast or ovarian cancer characterized by DNA repair deficiencies such as *BRCA1* and *BRCA2* mutations [47]. The undisputed advantage of the possible reuse of PARP inhibitors in other clinical settings is their well-characterized and favorable safety profiles in clinical trials, as well as the known side effects in the body [48]. Numerous open questions remain, particularly for the efficiency of prevention of LPS tolerance induction in in vivo and optimization of the optimal moment for PARP trap application. Currently, several models of septic shock induction have been described, however none of them are adequate to satisfy in sepsis research [49]. For example LPS injection, which has been used for nearly 100 years of sepsis studies, mimics most of the physiology of severe sepsis, but the doses of LPS to induce murine response are dissimilar from humans, which can be connected with different values of the median lethal dose (LD50) for LPS [50,51]. Explanation of those differences can be related to expression of various protective proteins among species or the use of LPS featured by different purity [52]. Other methods of sepsis induction in mice are also taken into consideration. They include the injection of bacteria or cecal contents from a donor rodent, the wildly used Cecal Ligation and Puncture (CLP) model, or colon ascendens stent peritonitis (CASP). These new methods in the murine model more adequately mimic dysfunction of organs observed in human sepsis [50]. Nevertheless, due to the existing differences among species, the physiological and immunological consequences of sepsis differ and limit the credibility of the conclusions, which drive the tested compounds or approaches to clinical trials [49].

In conclusion, the interaction of PARP1 with the promoter of *TNF-α* determines chromatin structure and its responsiveness to p65 recruitment upon activation of canonical NF-κB pathway with LPS. PARP traps emerge as efficient inhibitors of development of LPS-induced tolerance in the in vitro culture of human macrophages.

## 4. Materials and Methods

### 4.1. Materials

THP1 cells and human monocytes were purchased from American Type Culture Collection (ATCC) (Manassas, VA, USA) and from healthy donors from the blood bank in Lodz, respectively. RosetteSep™ Monocyte Enrichment Cocktail was purchased from STEMCELL Technologies (Grenoble, France); cell culture media were from Biowest (CytoGen, Zgierz, Poland); granulocyte–macrophage colony-stimulating factor (GM-CSF) from PeproTech (London, UK); BLUeye prestained protein ladder (#94964), oligonucleotides for real-time PCR were from Sigma Aldrich (Poznan, Poland); 5x HOT FIREPol EvaGreen qPCR Mix Plus (no ROX) (CytoGen, Zgierz Poland), ChiP grade antibodies anti-histone H3 (#4620), anti-PARP1 (#9532) and normal rabbit IgG (#2729) were from Cell Signaling Technology (LabJOT, Warsaw, Poland); TRI Reagent, anti-rabbit IgG (A0545) (whole molecule)–peroxidase antibody produced in goat were from Sigma Aldrich (Poznan, Poland); Lipofectamine RNAiMAX, Dynabeads™ Protein G, High-Capacity cDNA Reverse Transcription Kit, SuperSignal™ West Pico Chemiluminescent Substrate, OptiMem were from Thermo Fisher Scientific (Warsaw, Poland). Lipopolysaccharide (LPS), PARG inhibitor—ADP-HPD (Dihydrate, Ammonium Salt–Calbiochem) were from Sigma Aldrich (Poznan, Poland), Olaparib, Niraparib (MK-4827) and Veliparib (ABT-888), MG-132 and iPARG were from Cayman Chemical Biokom, (Warsaw, Poland). ViaFect™ Transfection Reagent was purchased from Promega (Warsaw, Poland). The human PARP1 Gene cDNA Clone (full-length ORF Clone), expression-ready, untagged (HG11040-UT; pCMV3-PARP1), and pCMV3-untagged Negative Control Vector (CV011; pCMV3-EMPTY) were purchased from Hölzel Diagnostika Handels GmbH (Köln, Germany). Puromycin dihydrochloride, PARP-1 shRNA Plasmid (h) (sc-29437-SH) and Control shRNA Plasmid-A(sc-108060), antibodies: anti-p50 (sc-1190), anti-p65 (sc-372) were from Santa Cruz Biotechnology (Lodz, Poland).

### 4.2. Monocyte Isolation, Differentiation of Macrophages and Cell Culture

Human monocytes were isolated from buffy coats from healthy donors using RosetteSep Monocyte Enrichment Cocktail, as described previously [16]. After attachment to the plate, monocytes were differentiated with GM-CSF (5 ng/mL) for 7 days in RPMI with 10% FBS, penicillin/streptomycin (50 U/mL, and 50 μg/mL, respectively).

THP1 cells (monocytic leukemia/premonocytes) were cultured under the same conditions.

### 4.3. Induction of Immune Paralysis

To initiate the development of immune tolerance to bacterial endotoxin, differentiated macrophages were stimulated with a single dose of LPS for 24 h. The macrophage activating bacterial compound was present in the cell culture medium during the entire period of the tolerance induction and was not washed out before another round of macrophage treatment with LPS. The second dose aimed to test cell pro-inflammatory response and the mRNA level of pro-inflammatory cytokines was measured 2 h after cell activation.

To initiate the development of immune paralysis, human macrophages were subjected to the following doses of the TLR4 ligand: 0.01 ng/mL, 0.1 ng/mL, 1 ng/mL, and 10 ng/mL for 24 h, whereas for testing responsiveness, cells were stimulated with 10 ng/mL LPS for 2 h. After initial evaluation of the paralyzing potential, one combination of LPS concentrations (1 ng/mL for tolerance induction + 10 ng/mL for the second activation) was chosen and applied to experimental procedures.

The scheme of THP1 cells remained the same, but the first dose of LPS was set as 50 ng/mL.

To estimate the impact of PARP, PARG and NF-κB inhibitors on the tolerance development, these compounds were added to cells for 1 h prior to first cell stimulation with bacterial endotoxin. PARP inhibitors were used in the following concentrations: olaparib (1 µM), MK-4827 and ABT-888 (0.5 µM, 2.5 µM), whereas a PARG inhibitor, ADP–HPD (Dihydrate, Ammonium Salt—Calbiochem), and an inhibitor of the proteasome/NF-κB pathway, MG132, at concentrations of 10 µM and 1 µM, respectively.

### 4.4. Quantification of the Gene Expression

For mRNA quantification, total RNA was extracted with TRI Reagent™ and reverse-transcribed with High-Capacity cDNA Reverse Transcription Kit, and cDNA fragments were amplified by real-time PCR with 5x HOT FIREPol EvaGreen qPCR Mix Plus (no ROX). Primer pairs used for cDNA amplification are listed in Table 1. Analysis of gene expression was performed on the basis of the fold-change and *p*-values, and expression levels of ACTB and GAPDH were taken for normalization.

### 4.5. Western Blot

For PARP1 protein detection by western blot, cell lysates were separated by SDS-PAGE, transferred to nitrocellulose membranes, blocked with skimmed milk and stained overnight at 4 °C with primary rabbit anti-PARP1 antibody (1:1000), and next day by 1 h with secondary goat anti-rabbit HRP-conjugated antibody (1:10,000). The HRP-derived signal was developed using SuperSignal™ West Pico Chemiluminescent Substrate. Pictures were acquired with ChemiDoc-IT2 (UVP, Meranco, Poznan, Poland). Tubulin was used as a loading control.

### 4.6. Chromatin Immunoprecipitation (CHIP) Assay

The PARP1 occurrence at the *TNF-α* promotor was analyzed in THP1 cells stimulated with the single dose (50 ng/mL) of LPS for 1 h. In brief, the cells were fixed with 1% formaldehyde, quenched with 125 mM glycine and rinsed 3x with cold PBS. After centrifugation, the acquired pellet was lysed by lysis buffers with protease inhibitor cocktail and sonicated with the ultrasonic homogenizer Bandelin Sonopuls (HD 2070). Lysates were added to 1% Triton-X100 and centrifuged, and supernatants were incubated with antibody-conjugated magnetic beads (Dynabeads™ Protein G) at 4 °C overnight. The next day, the immunoprecipitated chromatin was washed and de-crosslinked overnight at 65 °C. The DNA was isolated with phenol/chloroform/isoamyl alcohol and analyzed via real-time PCR.

To identify the NF-κB binding site, we searched through the binding motifs for p65 and p50 in the proximal promoter of TNF-α. The window for analysis was set at ±1 kbp. The sequence derived from University of California, Santa Cruz (UCSC), Santa Curz, CA, USA. Genome Browser was processed with TFBind, and the following binding site was identified with parameters: p50: *p* = 0.789871, in position 28 on input sequence (+); p65: *p* = 0.783234, in position 27 on input sequence (+). Therefore, primers to TNF-α promoter at −305/−254 from the CDS region designed as follows: 5′-ACTACCGCTTCCTCCAGATGA-3′ (forward) and 5′-GGGAAAGAATCATTCAACCAGCGG-3′ (reverse).

### 4.7. Permanent Gene Silencing and Transient Overexpression

PARP1 stable knockdowns and corresponding controls were generated using Amaxa^®^ Nucleofector^®^ Technology in the Laboratory of Transcriptional Regulation, Institute for Medical Biology, PAS, Lodz, and with the kind help of prof. Łukasz Pułaski. In brief, 0.5 µg of PARP1 shRNA Plasmid and Control shRNA Plasmid-A were mixed with THP1 cells suspended in Nucleofector Solution, subjected to electroporation with Amaxa^®^ Nucleofector^®^ according to the manufacturer’s instructions, and immediately diluted with warm RPMI with 10% FBS. After 24 h in culture, cells were selected with puromycin (5 µg/mL) for a month. After selection, puromycin was added to cells every second week.

The transient PARP1 overexpression was carried out as described previously [53]. In brief, THP1 cells at a density of 1,000,000/mL were treated with the complexes of pCMV3-EMPTY or pCMV3-PARP1 vectors and transfection reagent ViaFect. After 24 h, cells were cultured for 24 h as described in 2.2 and then subjected to LPS treatment (±olaparib).

### 4.8. Statistical Analysis

Data are shown as mean ± standard deviation of the mean (SEM). One-way analysis of variance (ANOVA) was carried out in GraphPad Prism 5 to compare means in several groups and followed by the Tukey test to detect statistically significant differences between means (marked with * when *p* < 0.05).

## Figures and Tables

**Table 1:** Sequences (5′–3′) of primers used.

GENE	FORWARD	REVERSE
*TNF-α*	GGAGAAGGGTGACCGACTCA	TGCCCAGACTCGGCAAAG
*ACTB*	TGGCACCCAGCACAATGAA	CTAAGTCATAGTCCGCCTAGAAGCA
*GAPDH*	TTCTTTTGCGTCGCCAGCCGA	GTGACCAGGCGCCCAATACGA
*COX2*	GAATCATTCACCAGGCAAATTG	TGGAAGCCTGTGATACTTTCTGTACT
*MIP2a*	CGCCCAAACCGAAGTCAT	GATTTGCCATTTTTCACATCTTT
*PARP1*	AAGCCCTAAAGGCTCAGAACG	ACCATGCCATCAGCTACTCGGT
*IL6*	GGCACTGGCAGAAAACAACC	GCAAGTCTCCTCATTGAATCC
*IL12a*	CTCCTGGACCACCTCAGTTTG	GGTGAAGGCATGGGAACATT
*iNOS*	GTTCTCAAGGCACAGGTCTC	GCAGGTCACTTATGTCACTTATC

## Data Availability

Raw data will be shared upon request.

## Supplementary Materials

The following are available online at https://www.mdpi.com/1424-8247/14/2/170/s1, Figure S1: The effect of human monocyte treatment with 1μM Olaparib for 1 h prior to induction of tolerance on the transcription of TNFα that was measured by real-time PCR, Figure S2: The effect of human macrophage treatment with PARP1 inhibitors 1 h on the tran-scription of TNFα that was measured by real-time PCR; Abbreviations: Olap—Olaparib, Velip—Veliparib, Nirap—Niraparib, LPS—lipopolysaccharide, Figure S3: The representative full length western blot images of ADP-ribosylated proteins and H3; cropped, red rectangular indicate picture areas that are included in the main Figures, Figure S4: The representative full length western blot images of PARP1 and H3 in the PARP1 sta-ble knock-downs; cropped, red rectangular indicate picture areas that are included in the main Figures, Figure S5: The representative full length western blot images of PARP1 and H3 in cells transiently transfected with the PARP1 expressing vector; cropped, red rectangular indicate picture areas that are included in the main Figures.
