# Supplementary material for: PARP Traps Rescue the Pro-Inflammatory Response of Human Macrophages in the In Vitro Model of LPS-Induced Tolerance
Source: Pharmaceuticals (Basel). 2021 Feb 22;14(2):170. doi: 10.3390/ph14020170 (PMC7926882; doi:10.3390/ph14020170)
Supplement: Supplementary file 1 [file pharmaceuticals-14-00170-s001.pdf]

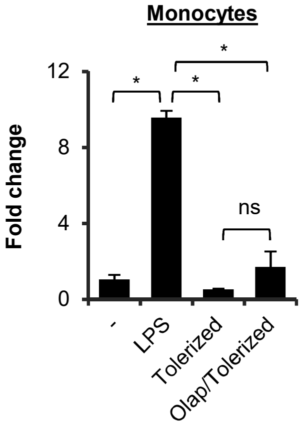

Figure S1: The effect of human monocyte treatment with 1μM Olaparib for 1 h prior to induction of toler-ance on the transcription of TNFα that was measured by real-time PCR

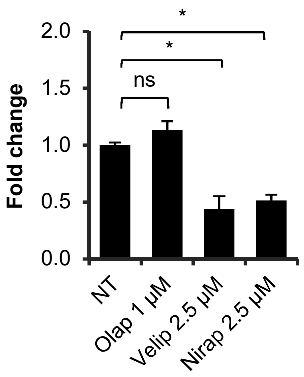

Figure S2: The effect of human macrophage treatment with PARP1 inhibitors 1 h on the tran-scription of TNFα that was measured by real-time PCR; Abbreviations: Olap—Olaparib, Velip—Veliparib, Nirap—Niraparib, LPS—lipopolysaccharide

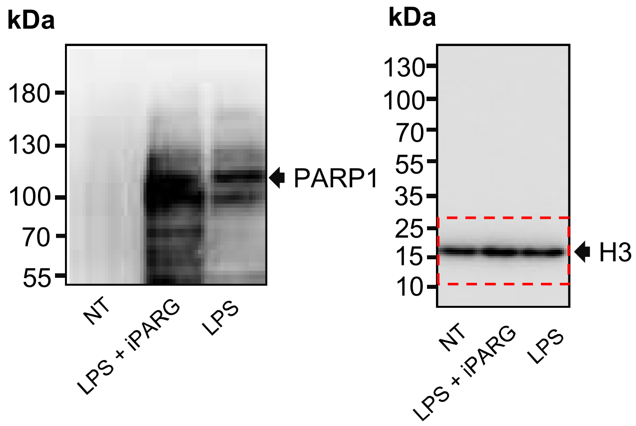

Figure S3: The representative full length western blot images of ADP-ribosylated proteins and H3; cropped, red rectangular indicate picture areas that are included in the main Figures.

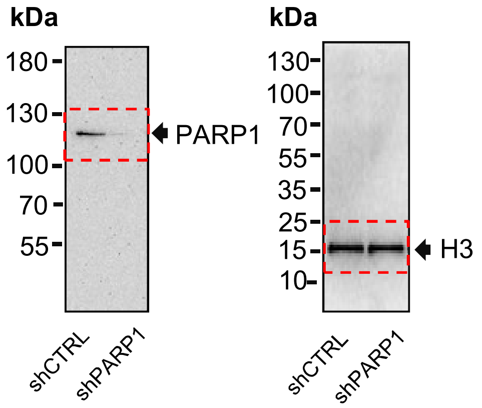

Figure S4: The representative full length western blot images of PARP1 and H3 in the PARP1 sta-ble knock-downs; cropped, red rectangular indicate picture areas that are included in the main Figures

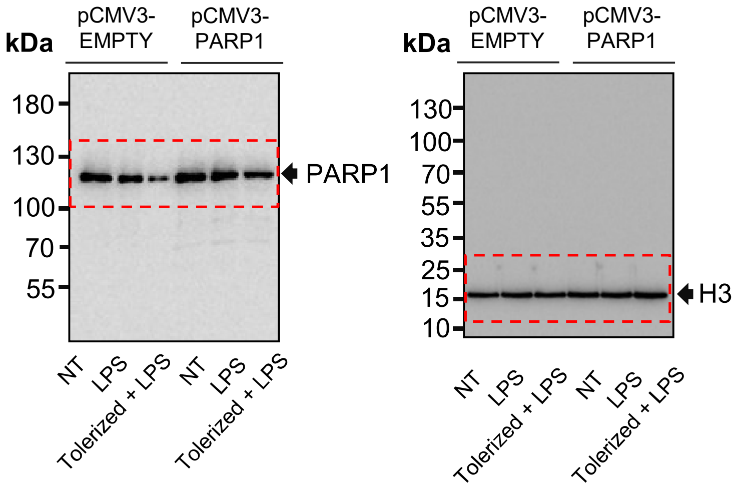

Figure S5: The representative full length western blot images of PARP1 and H3 in cells transiently transfect-ed with the PARP1 expressing vector; cropped, red rectangular indicate picture areas that are in-cluded in the main Figures
